# Supplementary material for: The Experiences of Informal Carers during the COVID-19 Pandemic: A Qualitative Systematic Review
Source: Int J Environ Res Public Health. 2022 Oct 18;19(20):13455. doi: 10.3390/ijerph192013455 (PMC9603569; doi:10.3390/ijerph192013455)
Supplement: Supplementary file 1 [file ijerph-19-13455-s001.zip › Figure S1-Mapping of codes and themes.pdf]

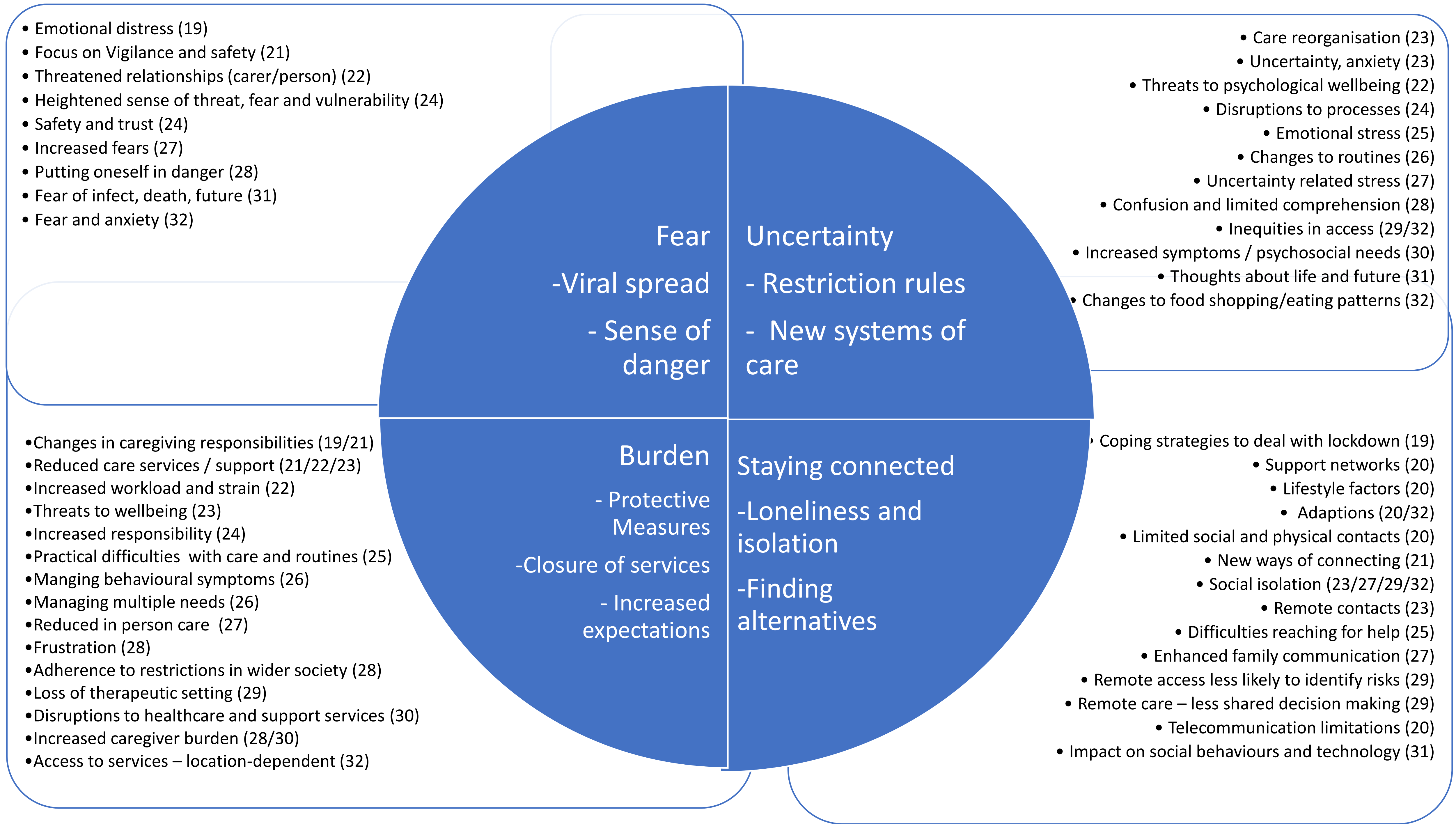
Emotional distress (19)
Focus on Vigilance and safety (21)
Threatened relationships (carer/person) (22)
Heightened sense of threat, fear and vulnerability (24)
Safety and trust (24)
Increased fears (27)
Putting oneself in danger (28)
Fear of infect, death, future (31)
Fear and anxiety (32)
Fear
-Viral spread
- Sense of danger
Uncertainty
- Restriction rules
- New systems of care
Care reorganisation (23)
Uncertainty, anxiety (23)
Threats to psychological wellbeing (22)
Disruptions to processes (24)
Emotional stress (25)
Changes to routines (26)
Uncertainty related stress (27)
Confusion and limited comprehension (28)
Inequities in access (29/32)
Increased symptoms / psychosocial needs (30)
Thoughts about life and future (31)
Changes to food shopping/eating patterns (32)
Changes in caregiving responsibilities (19/21)
Reduced care services / support (21/22/23)
Increased workload and strain (22)
Threats to wellbeing (23)
Increased responsibility (24)
Practical difficulties with care and routines (25)
Manging behavioural symptoms (26)
Managing multiple needs (26)
Reduced in person care (27)
Frustration (28)
Adherence to restrictions in wider society (28)
Loss of therapeutic setting (29)
Disruptions to healthcare and support services (30)
Increased caregiver burden (28/30)
Access to services – location-dependent (32)
Burden
- Protective Measures
-Closure of services
- Increased expectations
Staying connected
-Loneliness and isolation
-Finding alternatives
Coping strategies to deal with lockdown (19)
Support networks (20)
Lifestyle factors (20)
Adaptions (20/32)
Limited social and physical contacts (20)
New ways of connecting (21)
Social isolation (23/27/29/32)
Remote contacts (23)
Difficulties reaching for help (25)
Enhanced family communication (27)
Remote access less likely to identify risks (29)
Remote care – less shared decision making (29)
Telecommunication limitations (20)
Impact on social behaviours and technology (31)
